# Corrosion Inhibition of Expired Cefazolin Drug on Copper Metal in Dilute Hydrochloric Acid Solution: Practical and Theoretical Approaches

**DOI:** 10.3390/molecules29051157

**Published:** 2024-03-05

**Authors:** Raiedhah A. Alsaiari, Medhat M. Kamel, Mervate M. Mohamed

**Affiliations:** 1 Empty Quarter Research Centre, Department of Chemistry, Faculty of Science and Arts in Sharurah, Najran University, Sharurah 78362, Saudi Arabia; raalsayari@nu.edu.sa (R.A.A.); mervate71@gmail.com (M.M.M.); 2 Department of Chemistry, Faculty of Science, Suez Canal University, Ismailia 41522, Egypt

**Keywords:** expired Cefazolin, copper, corrosion inhibition, hydrochloric acid

## Abstract

In this work, we studied the corrosion of Cu metal in 0.5 mol L^−1^ HCl and the inhibition effect of the expired Cefazolin drug. The inhibition efficiency (IE) of Cefazolin varied according to its concentration in solution. As the Cefazolin concentration increased to 300 ppm, the IE increased to 87% at 298 K and decreased to 78% as the temperature increased to 318 K. The expired drug functioned as a mixed–type inhibitor. The adsorption of the drug on the copper surface followed Temkin’s adsorption model. The magnitudes of the standard free energy change (Δ*G^o^_ads_*) and adsorption equilibrium constant (*K_ads_*) indicated the spontaneous nature and exothermicity of the adsorption process. Energy dispersive X-ray (EDX) and scanning electron microscopy (SEM) techniques showed that the drug molecules were strongly attached to the Cu surface. The electrochemical frequency modulation (EFM), potentiodynamic polarization (PP), and electrochemical impedance spectroscopy (EIS) results were in good agreement with the results of the weight loss (WL) method. The density functional tight–binding (DFTB) and Monte Carlo (MC) simulation results indicated that the expired drug bound to the copper surface through the lone pair of electrons of the heteroatoms as well as the π-electrons of the tetrazole ring. The adsorption energy between the drug and copper metal was –459.38 kJ mol^−1^.

## 1. Introduction

Corrosion negatively affects the performance of materials and structures. It causes most metallic structures to lose strength, performance, and appearance [1]. Corrosion has a significant harmful economic impact on different businesses and negative environmental repercussions. Corrosion contributes to operational risks and apparatus maintenance, leading to frequent partial and even complete process termination, which causes significant economic damage [2,3].

According to studies conducted all over the world, corrosion damage is extremely high; utilizing the right corrosion control techniques, such as coating, painting, using expensive alloys, and applying inhibitors, can reduce the total damage by 20–25% [4,5]. Inhibitors are frequently used in acidic media to prevent corrosion of the metals [6]. Under various circumstances, inhibitors are beneficial for the preservation of metals. The mode of action of the inhibitor accounts for this selectivity. Inhibitors are especially useful in closed systems where the harmful environment is recycled or held for some time [7,8,9,10].

Salt hydrolysis causes acid formation during crude oil cracking. This will cause a disastrous impact on machinery. Inhibitors are powerful tools to eliminate this damaging effect. To prevent the corrosion of metals in acid electrolytes, organic inhibitors are most frequently used [11,12,13,14].

Cu and its alloys offer superior qualities for various applications. They have widespread applications in valve systems, condensers, and exchangers. Also, they are employed in the architectural industry for objects such as doorknobs, rods, padlocks, and building fronts. Copper is resistant to corrosion in different chemical media, but it is sensitive to corrosion in hydrochloric acid solutions [15]. Diluted HCl solutions are frequently used to remove scales and other corrosion products from industrial equipment [16,17,18].

The use of pharmaceuticals as corrosion inhibitors has recently increased [19]. Because of their eco–quality, these drugs are superior to common inhibitors. Drugs have replaced the traditional poisonous corrosion inhibitors because they are safe and have little detrimental environmental effects. A drug is employed as a corrosion inhibitor according to the following three criteria: (a) the drug structure has donor atoms, such as O, N, and S; (b) the drug is eco–friendly and useful in biological systems; and (c) the drug is easily prepared and purified. 

Undoubtedly, the bulk of pharmaceutical treatments are more expensive than the standard organic inhibitors used in the industry. Thus, it is economically useless to use a fresh curing agent as a corrosion inhibitor. Consequently, it makes sense to use the properties of expired drugs to limit corrosion. Even after their expiration dates, pharmaceuticals drugs keep at least 90% of their initial potency; however, due to professional constraints and liability concerns, their use was restricted for therapeutic purposes [20]. The use of expired drugs could overcome two more sensitive problems, e.g., reducing environmental pollution with drugs and decreasing drug disposal costs [21]. 

Scientists have recently employed expired drugs, such as Ampicillin [22], Antidiabetic [23], Herbal [24], Ampicillin and Flucloxacillin [25], Acyclovir and Omeprazole [26], Ceftin [27], Antihypertensive [28], Antifungal [29], 6–mercaptopurine and 6–thioguanine [30], Ciprofloxacin [31], Glucosamine [32], Flunarizine Hydrochloride [33], Amiodarone [34], Phenylphrine [35], Betamethasone [36], Podocip [37], Gentamycine [38], Lioresal [39], Midazolam [40], Fluoxymesterone [41], Atorvastin [42], etc., to inhibit the corrosion of different metals. 

Cefazolin, a wider cephalosporin antibiotic, is employed for moderate–to–intense bacterial infections of the lungs, bones, joints, blood, heart valves, and urinary system, and skin infections. When given parenterally and orally to mice and rats, the antibiotic Cefazolin has relatively low acute toxicity. Cefazolin has no effect on the reproductive system, or the progeny of rats treated before and during mating, gestation, or breastfeeding. It is not teratogenic for mice or rabbits [43]. Cefazolin has various binding sites, including N, S, and O atoms. The ability of the drug to inhibit corrosion occurs via interaction with the metal surface through the adsorption process. Unexpired Cefazolin is more expensive than the tested organic inhibitors. Therefore, the focus of this study was on the use of expired Cefazolin as a corrosion inhibitor. According to this work, the expired Cefazolin drug can decrease the cost of disposal and the requirement for environment friendly products. This study used electrochemical and chemical techniques to assess the corrosion inhibition of Cu in a 0.5 mol L^−1^ HCl solution using expired Cefazolin. The drug is cheap and non–toxic [43]. To the best of our knowledge, no one has ever proposed using outdated Cefazolin drug to inhibit copper dissolution in HCl media.

This work aimed to study the impact of the expired Cefazolin drug on copper metal corrosion in a 0.5 mol L^−1^ HCl pickling solution. EIS, WL, EFM, and PP techniques were used to conduct the experiments. SEM and EDX techniques examined the morphology and composition of the copper metal surface. Different adsorption isotherms were utilized to assess the drug’s adsorption type on copper metal. The thermodynamic parameters related to the adsorption process were also determined. DFTB, MC, and computational investigations described the interactions between the Cefazolin molecules and the copper metal surface.

## 2. Results and Discussion

### 2.1. WL Measurements

#### 2.1.1. The Impact of Drug Concentration

The corrosion rate (*k*) and inhibition efficiency were determined for blank and different concentrations (50–300 ppm) of the expired Cefazolin drug using the WL method. WL measurements were used to assess Cefazolin’s inhibition activity. Equation (1) is used to compute the corrosion rate (*k*) [44].

(1) $$k=\frac{\Delta W}{At}$$

where Δ*W* is the WL of the Cu sheet in mg, *A* is the area of the Cu sheet in cm^2^, and *t* is the immersion time in minutes. Equation (2) is used to calculate the inhibition efficiency (*IE_w_*) [45].

(2) $$IE_{w}=\left(1-\frac{W_{inh}}{W_{corr}}\right)\times 100$$

where *W*_corr_ and *W*_inh_ are the WL of Cu sheets without and with the drug, respectively. Table 1 shows WL data for copper metal in 0.5 mol L^−1^ HCl without and with different doses of the expired Cefazolin at different temperatures. The dissolution of copper to form the cuprous ion, Cu^+^, causes the anodic reaction of Cu in HCl acid to occur rapidly [46].

Cu → Cu^+^ + e^−^ (fast) (3)

The cuprous ion reacts with the chloride ion in solution, and forms the insoluble CuCl, which precipitates on the Cu surface [47,48].

Cu^+^ + Cl^−^ → CuCl (4)

The produced CuCl provides insufficient protection for the Cu surface because of its poor cohesion. It interacts with one other Cl^−^ ion to form the soluble chloride complex, CuCl_2_^−^.

CuCl + Cl^−^ → CuCl_2_^−^ (5)

The result is that copper corrodes. The soluble complex, however, could oxidize to cupric ions, Cu^2+^.

CuCl_2_^−^ → Cu^2+^ + 2Cl^−^+ e (6)

In oxygenated acidic chloride solutions, the cathodic reaction is:

4H^+^ + O_2_ + 4e → 2H_2_O (7)

Consequently, copper in acid chloride solutions exhibits the overall corrosion reaction:

2Cu + 4H^+^ + 4Cl^−^ + O_2_ → 2H_2_O + 4Cl^−^ + 2Cu^2+^ (8)

**Figure 1:**
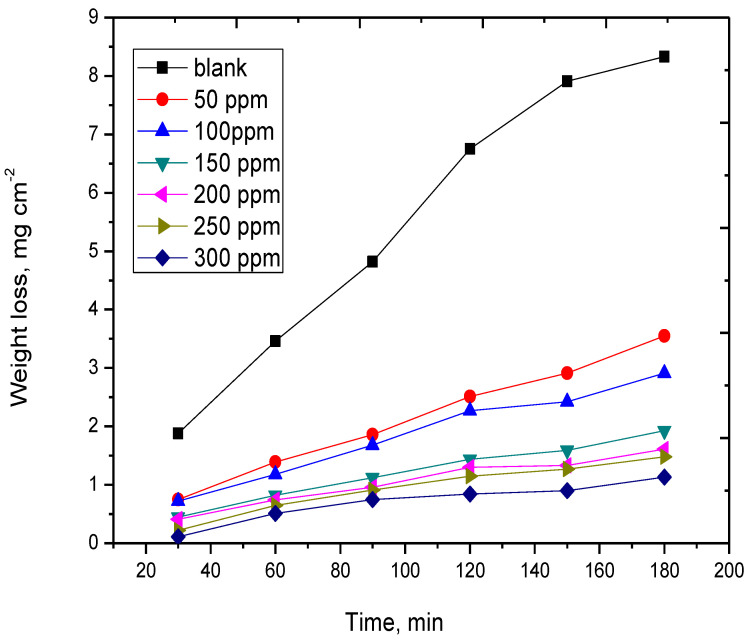
WL curves of copper metal in 0.5 mol L^−1^ HCl without and with distinct concentrations of expired Cefazolin at 298 K.

Contrarily, Figure 1 shows that the expired Cefazolin significantly decreases the WL of Cu metal in HCl solution. The adsorption of the drug molecules on the Cu surface suppresses the tendency of copper to dissolve and reduces the rate of corrosion. When the drug is present at a concentration of 300 ppm, *IE_w_* reaches 87% at 298 K. This clarifies that the expired Cefazolin may effectively inhibit the corrosion of Cu metal in HCl solution.

#### 2.1.2. Impact of Temperature on Inhibition Efficiency

It is quite challenging to foresee how temperature will affect the inhibited acid–metal reaction because of the different changes on the metal surface. These alterations can take different forms, including quick etching, desorption of the inhibitor, and the inhibitor’s own breakdown or restructuring [49]. Both *k* and *IE*_w_ are measured in the temperature range (298–318 K). Table 1 displays the impact of temperature on *IE* and *k*. In the presence or absence of different dosages of the expired antibiotic, the increase in temperature results in an increase in the corrosion rate of Cu metal. This could result from the increased impact of rising temperatures on the rate of electrochemical reactions.

According to the data in Table 1, *IE_w_* decreases as the temperature increases. Desorption of the drug molecules from the Cu surface will be the cause [50]. This result clearly shows that the studied drug attaches to the Cu surface in a physical form of adsorption since the rate of the desorption process increases with increasing temperature.

### 2.2. Adsorption Type and Chemical Thermodynamics Approach

The ability of heterocyclic compounds to adsorb on a metal surface and create a protective film is the main cause of the corrosion inhibition. The double–layer structure is disturbed by the interaction of anticorrosion molecules with the metal surface, which may cause the water molecules at the metal–electrolyte interface to reorganize as follows:

Drug_(soln.)_ + H_2_O_(ads)_ → Drug_(ads)_ + H_2_O_(soln.)_ (9)

**Figure 2:**
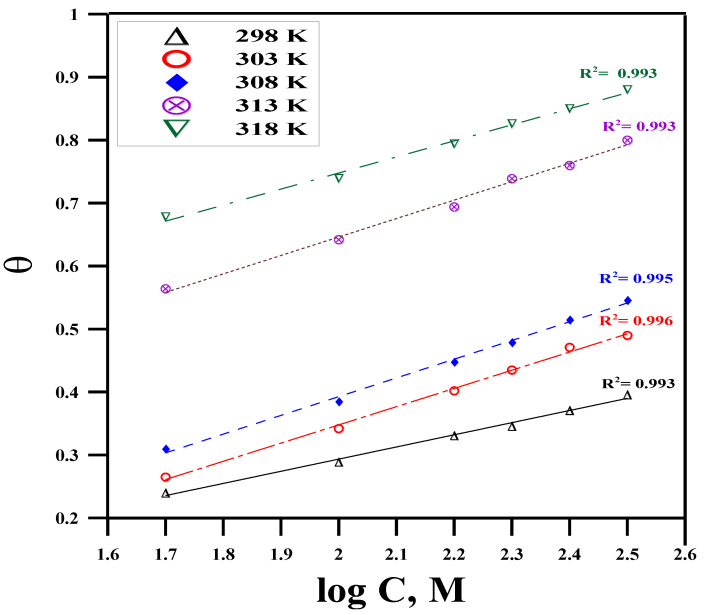
Temkin’s model at different concentrations of expired Cefazolin at different temperatures for copper metal in 0.5 mol L^−1^ HCl solution.

**Figure 3:**
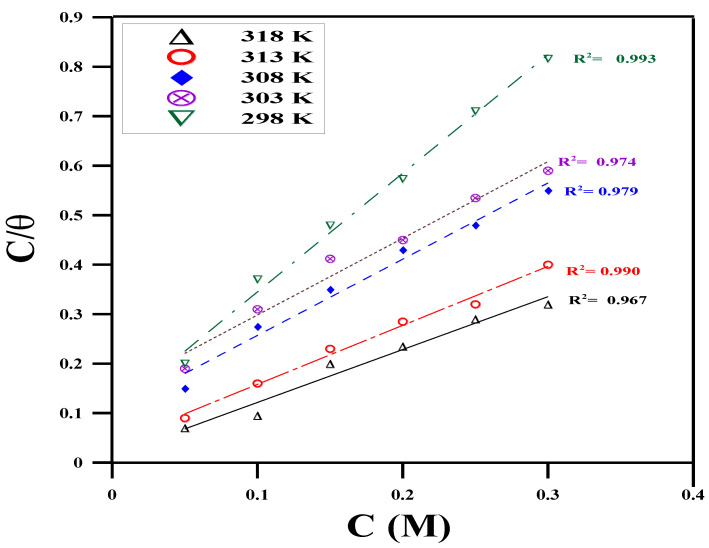
Langmuir’s model at different concentrations of expired Cefazolin at different temperatures for copper metal in 0.5 mol L^−1^ HCl solution.

**Figure 4:**
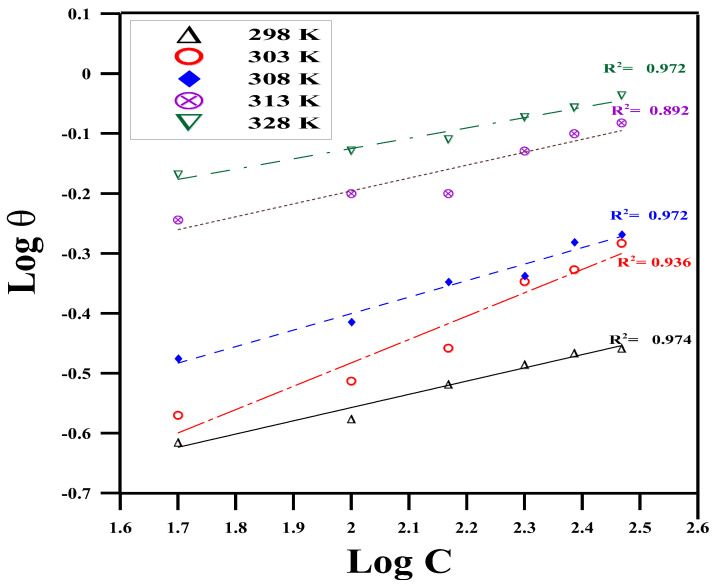
Freundlich’s model at different concentrations of expired Cefazolin at different temperatures for copper metal in 0.5 mol L^−1^ HCl solution.

It is crucial to identify the isotherms that describe the adsorption behavior of corrosion inhibitors because it reveals the type of interaction between the metal and the inhibitor [51]. As a result, different adsorption isotherms, including Temkin, Langmuir, and Freundlich, are investigated (Figure 2, Figure 3 and Figure 4); the isotherm that best fits the surface coverage data is shown as the most representative. Typically, the highest regression coefficient *R*^2^ indicates the best fit. Plotting the surface covering (*θ*) data is performed using the measured WL data. Upon conducting a comprehensive analysis of the fitting of different adsorption isotherms, the Temkin model is found to exhibit the best linear regression, with *R*^2^ ≥ 0.991 (according to Figure 2) [52]. In this instance, the adsorption mechanism is best described by the Temkin isotherm.

According to Temkin’s model, Equation (10) correlates *θ_coverage_* to the equilibrium constant (*K_ads_*) for the adsorption process [53].

(10) $$\theta_{coverage}=\frac{2.303}{a}\;logK_{ads}+\frac{2.303}{a}logC$$

where *θ* is the fraction of surface coverage (*θ = IE_w_*/100), *K_ads_* is the equilibrium constant, *C* is the drug’s concentration, and “*a*” is a molecular interaction parameter. A straight line of a slope $\frac{2.303}{a}$ and an intercept of $\frac{2.303}{a}\;$*log* $K_{ads}$ are obtained by drawing a relationship between *θ* and *log C*.

Table 2 estimates and summarizes the *K_ads_* magnitudes. At lower temperatures, the values of *K_ads_* are high, indicating that the drug is strongly adsorbed on the Cu surface. The desorption rate of the antibiotic molecules increases with increasing temperature, which causes the values of *K_ads_* to decrease. The standard Gibbs free energy (Δ*G^o^_ads_*), which is derived from Equation (11), is another parameter related to the adsorption constant (*K_ads_*) [54,55].

(11) $$\Delta G_{ads}^{o}=-RT\;\ln\left(55.5\;K_{ads}\right)$$

The estimated values of Δ*G^o^_ads_* are between −12.81 and −16.68 kJ mol^−1^, showing that the drug is physically adsorbed on copper metal at the temperatures used. The finding that *IE_w_* decreases with increasing temperature supports this conclusion. The negative magnitudes of Δ*G^o^_ads_* signify the stable state of the adsorbed film on the CS surface as well as the spontaneous action of the separated molecules during the adsorption process [56]. The presence of heteroatoms and π–electrons in the structure of the expired drug may be responsible for its interaction with the copper surface.

### 2.3. PP Measurements

**Figure 5:**
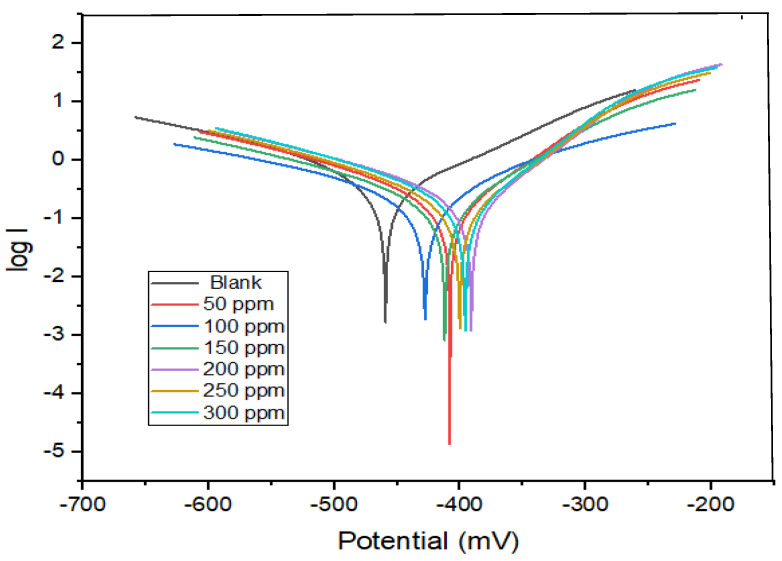
PP for the corrosion of copper in 0.5 mol L^−1^ HCl without and with different concentrations of expired Cefazolin at 298 K.

Figure 5 shows copper polarization curves in 0.5 mol L^−1^ HCl without and with distinct dosages of the antibiotic Cefazolin. The corrosion current (*i_corr_*) markedly decreases as the Cefazolin concentration increases. Therefore, the antibiotic can successfully inhibit copper metal corrosion in an acidic medium. The cathodic polarization curves in Figure 5 have a consistent trend, showing that Cefazolin’s adsorption on the copper surface has no effect on the cathodic reaction mechanism. In the presence of Cefazolin, the slopes of the anodic curves have changed, suggesting that the mechanism of the anodic reaction has altered. The Tafel extrapolation method is used to determine *E_corr_* and *i_corr_* [12]. The results are shown in Table 3. Equation (12) is used to calculate the corrosion current, *i_corr_*.

*i_corr_* = *B/R_p_* (12)

where *R_p_* is the polarization resistance and *B* is a constant related to Stern and Geary. *B* is calculated according to the relationship:

(13) $$B=\frac{\beta_{a}\cdot \;\;\beta_{c}}{2.303\left(\beta_{a}+\beta_{c}\right)}$$

where *β_a_* and *β_c_*, respectively, are the anodic and cathodic Tafel constants. The following equation is used to compute *IE_p_*.

(14) $$IE_{p}=\left(1-\frac{i_{\;\left(inh\right)}}{i{\;}_{\left(free\right)}}\right)\times 100$$

*i_(free)_* and *i_(inh)_* are the corrosion current densities in the absence and presence of the drug, respectively. In accordance with Table 3, *E_corr_* is −420 mV (SCE) for the blank solution. The corrosion potential changes to a less negative value after the addition of Cefazolin, but without a clear trend. This indicates that Cefazolin functions as a mixed–type inhibitor in 0.5 mol L^−1^ HCl, forming an adsorbed film at the metal/solution boundary that decreases the available anodic and cathodic sites. Cefazolin antibiotic serves as a mixed–type corrosion inhibitor as the maximum change in corrosion potential caused by the drug is less than 85 mV [57]. This indicates that both half–reactions (anodic and cathodic) are altered by the addition of the expired drug. The cathodic Tafel slopes change greater than the anodic Tafel slopes, implying that Cefazolin molecules are adsorbed on both sites but preferentially under cathodic control, which inhibits the cathodic reduction reaction and anodic dissolution [56].

### 2.4. EIS Study

EIS provides considerable mechanistic and kinetic details for the electrochemical system under investigation. EIS measures the ability of a circuit to counteract the flowing electrical current. It is established by exposing the electrochemical cell to an AC potential and measuring the result in the cell. EIS offers an alternative technique for describing the adsorbed film on the electrode that is illustrated by the charge transfer resistance (*R_ct_*). It has been shown that the interface capacitance can be used to evaluate the film quality [58].

**Figure 6:**
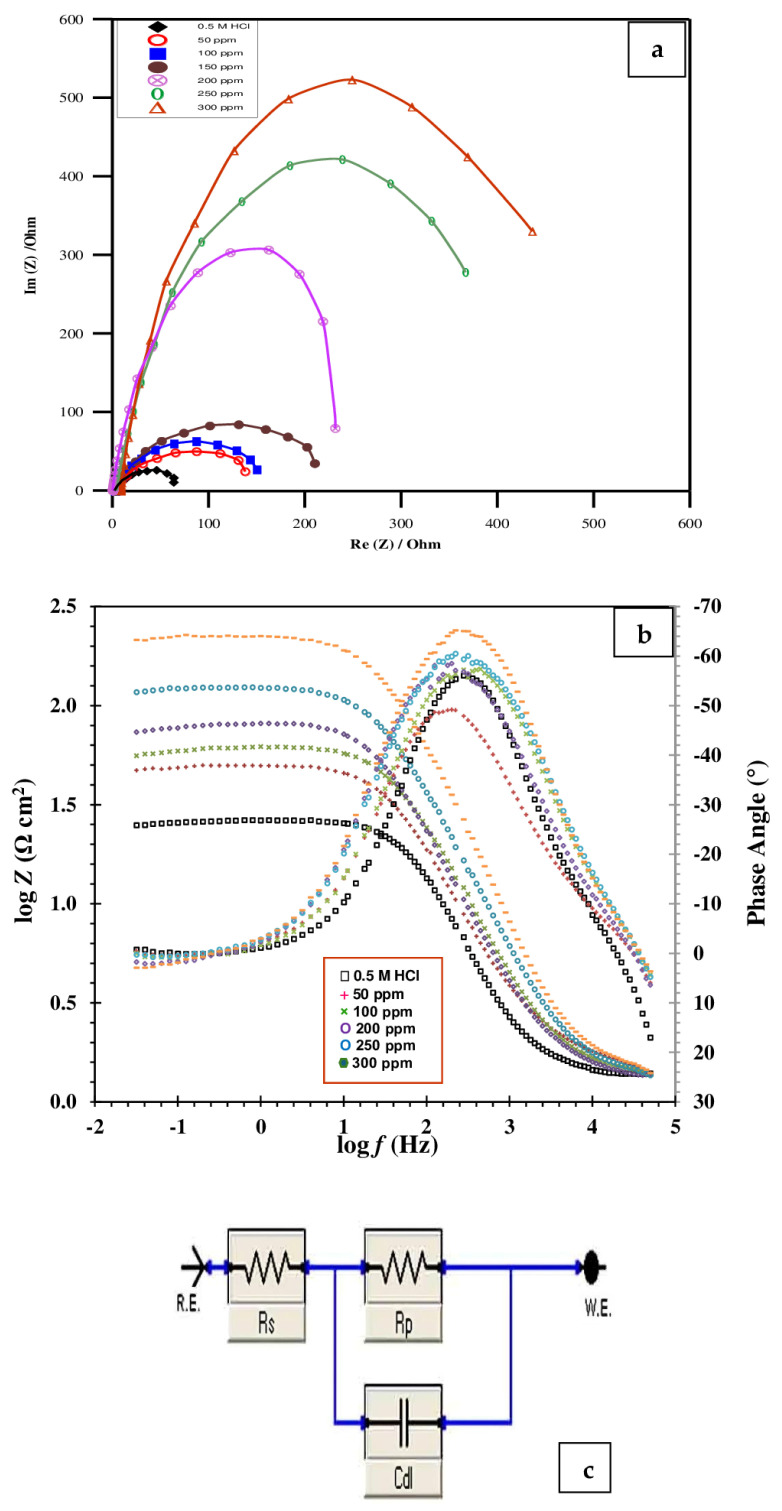
Nyquist (**a**) and Bode (**b**) plots and electrochemical equivalent circuit (**c**) for the corrosion of copper in 0.5 mol L^−1^ HCl without and with different concentrations of expired Cefazolin at 298 K.

Figure 6a,b displays the Nyquist and Bode plots for the Cu in 0.5 mol L^−1^ HCl with different Cefazolin dosages at 298 K. The impedance spectra of copper have a low–frequency inductive loop and a high–frequency capacitive loop, except at concentrations greater than 200 ppm. The time constant and charge transfer reaction of the electrical double layer is responsible for the high–frequency capacitive loop. At high frequencies, the surface film formation could be the cause of the time constant. However, the inductive loop has been related to a disintegration process, bulk relaxation process, or surface relaxation process [56]. The relaxation process caused by the adsorption of species like H_ads_ and Cl^−^ on the copper surface may be the cause of the low–frequency inductive loop in the inhibited acid solution containing a lower Cefazolin concentration. It could possibly be related to the passivated surface’s re–dissolution at low frequencies. Our opinion is supported by the fact that this semicircle disappears when a higher concentration of Cefazolin is added [56]. The diameter of the capacitance loop increases significantly as the dose of the Cefazolin drug increases in the corrosive medium (Figure 6a). This indicates that the antibiotic Cefazolin, which has a considerable inhibitory effect on the corrosion of copper, adsorbs and increases the charge transfer resistance (*R_ct_*). As the concentration of the drug increases in the solution, the semicircle’s diameter grows larger. This demonstrates that the electron transfer step is the slowest step throughout the corrosion process. Regarding what the EIS theory predicted, the loops in the high–frequency range are not idealized semicircles. This discrepancy may be attributed to the unsatisfactory performance of the double layer as a capacitor.

The imperfect effectiveness is assigned to the frequency dispersion, which is caused by the roughness and non–homogeneity of the surface.

The capacitive reactance modulus in the Bode plot increases by an order of magnitude in the low-frequency zone, as displayed in Figure 6b. Additionally, the slope of the impedance modulus is nearly one, and the phase angle attains −65° in the domain of intermediate frequency. This implies that the copper capacitance feature of Cefazolin is indeed present. When Cefazolin adsorbs on the surface of copper, it only creates a time constant, as can be observed in Figure 6b, where only one peak can be seen inside the phase angle. Figure 6c depicts the analogous Randles circuit. The circuit shows that the double–layer capacitance *C_dl_* and *R_ct_* are linked in parallel. Both outputs are linked in series with the solution resistance, *R_s_*. The practical findings are in good accord with this concept. Equation (15) is utilized to estimate *IE_s_* using *R_ct_* [59,60].

(15) $$IE_{s}=\frac{R_{ct}-R_{ct}^{o}}{R_{ct}}\times 100$$

where $R_{ct}^{o}$ and $R_{ct}$, respectively, stand for charge transfer resistance without and with the drug. The impedance characteristics are listed in Table 4. As the antibiotic concentration increases, *R_ct_* increases; however, *C_dl_* decreases. The antibiotic molecules gradually replace the water molecules at the Cu surface. As a result, the dissolution response will be minimized [61]. The dissolution of the metal slows down as the *R_ct_* values increase [53].

The reduction in *C_dl_* values is attributed to a decrease in the dielectric constant, other than the increasing thickness of the electrical double layer. This finding suggests that the drug molecules work at the metal surface through adsorption [62]. The interaction of the antibiotic with previously adsorbed Cl^−^ ions causes the adsorption to take place. As an alternative, the interaction could occur between the π–electrons of antibiotic molecules or unpaired electrons and the vacant orbitals of the metal [63].

### 2.5. EFM Study

**Figure 7:**
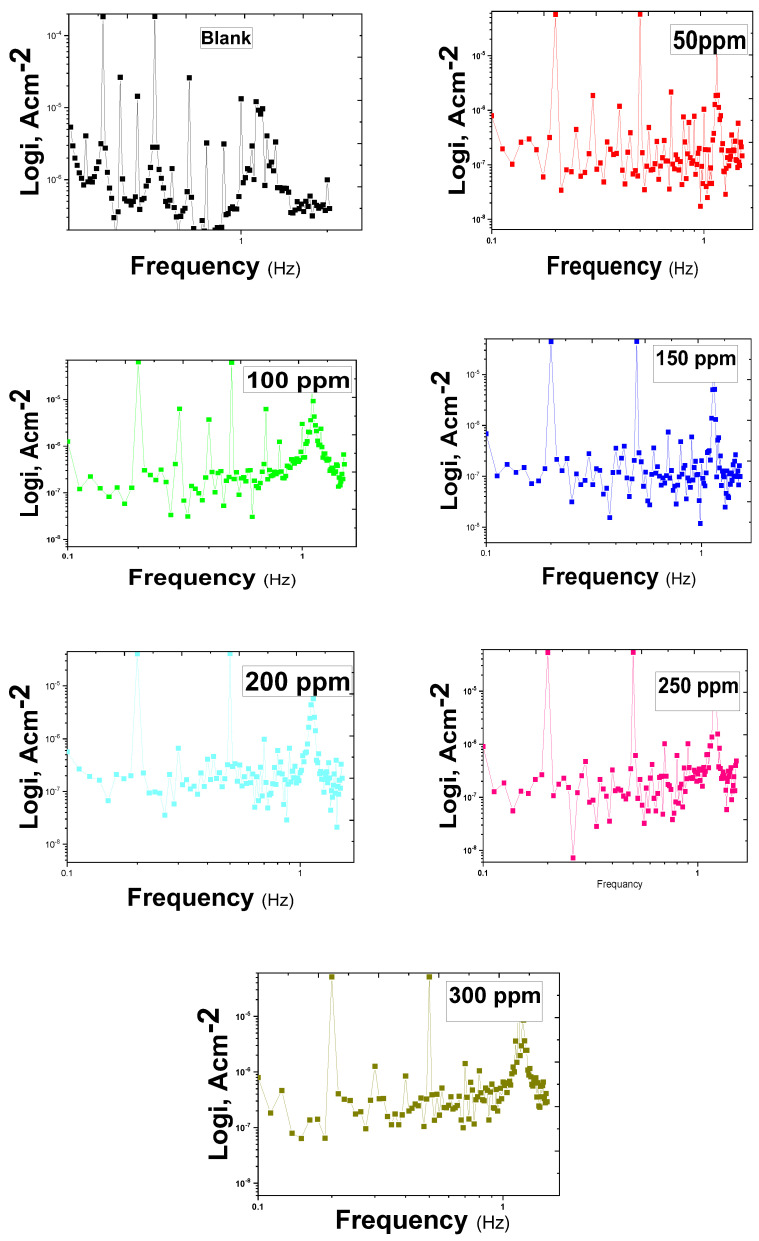
EFM spectra for the corrosion of copper in 0.5 mol L^−1^ HCl without and with distinct concentrations of expired Cefazolin at 298 K.

It is possible to calculate the instantaneous rate of corrosion by using the EFM technique. This can be achieved with a small polarizing signal and without previous knowledge of the Tafel characteristics. The EFM technique can track variations in the environment’s corrosiveness, such as changes in oxygen content and hydrodynamic circumstances. The EFM approach is a prime choice for online corrosion monitoring applications because of its benefits, such as direct nondestructive rapid measurement of corrosion rates and data validation [45]. Figure 7 shows the modulation spectra obtained from EFM testing for the dissolution of copper in a 0.5 mol L^−1^ HCl solution without and with different doses of expired Cefazolin. The current response includes both the input frequencies and frequency components. The latter results from the multiples, differences, and sum of the two input frequencies. As a result of the excitation frequencies, there are two main peaks at 0.2 and 0.5 Hz.

The Tafel slopes, the corrosion current, and the causality factors (CF–2, CF–3) are computed using the two main peaks. The peaks between 1 and 20 µA indicate the harmonics, summation, and differences between the two excitation frequencies. Table 5 displays the corrosion kinetic characteristics, including *IE_EFM_*, *i_corr_*, Tafel parameters, and causality factors. The corrosion current density decreases as the drug concentration increases. As the concentration of Cefazolin increases, the effectiveness of the inhibition increases. The causality factors are quite close to their predicted ones (Table 5). According to EFM theory, this could guarantee the accuracy of the Tafel parameters and corrosion current densities. [45]. Equation (16) provides *IE_EFM_* calculations based on the EFM data.

(16) $$IE_{EFM}=\frac{i_{corr}^{{}^{\circ}}-i_{corr}}{i_{corr}^{{}^{\circ}}}\times 100$$

where $i_{corr}^{{}^{\circ}}$ and $i_{corr}$ are the corrosion current densities in the absence and presence of the drug, respectively.

### 2.6. SEM and EDX Studies

**Figure 8:**
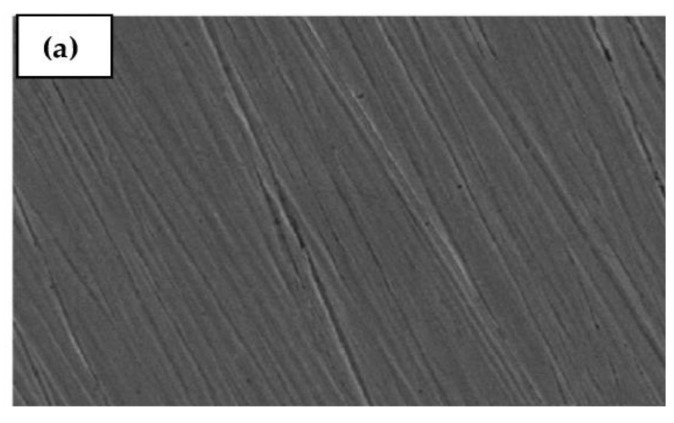

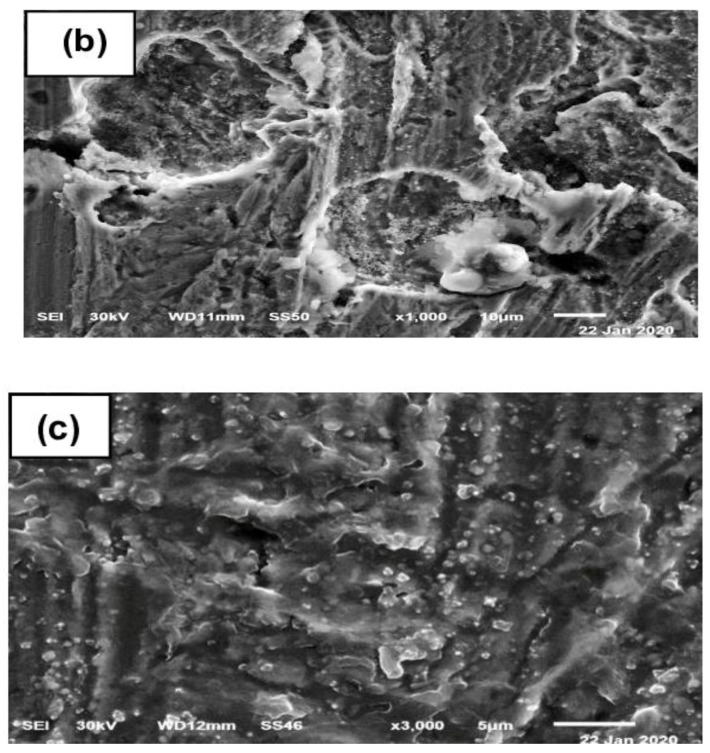
SEM micrographs of polishing copper (**a**), copper after 8 h of immersion in 0.5 mol L^−1^ HCl without (**b**) and with (**c**) 300 ppm of expired Cefazolin at 298 K.

The existence of a protective film of the expired drug on the Cu surface is further clarified by the SEM technique. Additionally, EDX studies are performed to check whether the drug is attached to Cu or not. SEM micrographs of the polished Cu and Cu immersed in a 0.5 mol L^−1^ HCl solution without and with 300 ppm of the Cefazolin antibiotic are shown in Figure 8. To initiate the corrosion process, many scratches are left on the surface of the polished copper, as shown in Figure 8a [26]. After being in contact with a 0.5 mol L^−1^ HCl solution for 8 h, the morphology of the copper surface is shown in Figure 8b. The entire Cu surface has deteriorated because of the pitting impact of the chloride ions in solution. As shown in Figure 8c, the expired drug effectively improves the surface morphology of copper because of its ability to form a protective film on the surface of copper metal.

**Figure 9:**
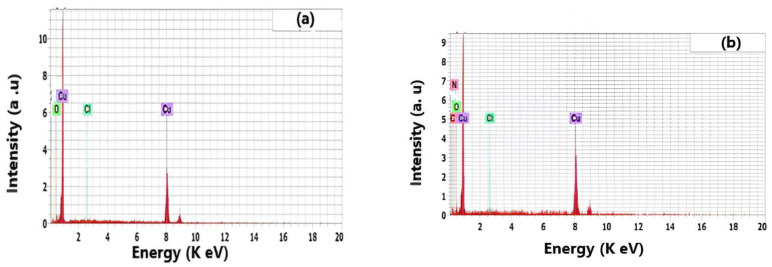
EDX analysis of copper after 8 h of immersion in 0.5 mol L^−1^ HCl without (**a**) and with (**b**) 300 ppm of Cefazolin at 298 K.

The presence of the drug molecules on the Cu surface is confirmed using the EDX technique. Figure 9 shows the corresponding EDX spectra. Both copper and chloride peaks can be seen for the samples that are exposed to the blank solution because CuCl_2_ is formed at the copper surface (Figure 9a). For samples that have been exposed to the expired drug, C, N, and O peaks can be observed in the EDX pattern (Figure 9b). This result reveals the adsorption of the drug molecules on the copper surface, showing a significant inhibition of the Cu corrosion. There is no peak for S. This indicates that the concentration of S is too small to be detected on the copper surface. The presence of the C, N, and O atoms on the metal surface suggests that the drug molecules are strongly adsorbed on the copper, forming a protective film. The formed film protects the copper from corrosion.

### 2.7. Quantum Chemical Calculations

#### 2.7.1. DFTB Results

Density functional tight–binding (DFTB) calculations can provide valuable information on the electronic and thermodynamic properties of corrosion inhibitors. The HOMO–LUMO energy gap (∆*E*) and other quantum chemical characteristics, such as the LUMO and HOMO energy levels, are utilized to explain the efficiency of Cefazolin’s inhibition.

The HOMO and LUMO energies are found to be −6.40 and −2.01, respectively, suggesting that the Cefazolin drug can function as an electron donor, which facilitates the formation of a protective film on the copper surface. The calculated Δ*E* value of 4.39 shows that Cefazolin has good stability and can effectively inhibit the corrosion process (Table 6).

**Figure 10:**
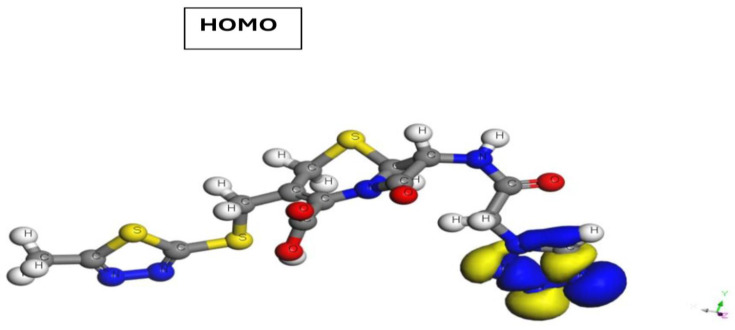

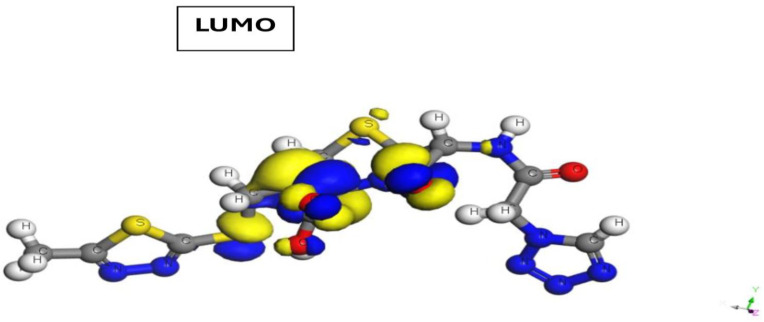
Molecular orbitals: HOMO and LUMO of Cefazolin drug.

According to the distribution of the HOMO electron density, the drug molecule can donate electrons to the Cu metal through the adsorption sites of the tetrazole ring (a five–membered ring made up of four nitrogen atoms and one carbon atom), as well as π–orbitals and hetero N atoms (Figure 10). However, the distribution of the LUMO electron density suggests that the drug molecule can accept electrons from the copper metal via the dithiazole ring and 5–thia–1–azabicyclo[4.2.0]oct–2–ene–2–carboxylic acid [64,65].

**Figure 11:**
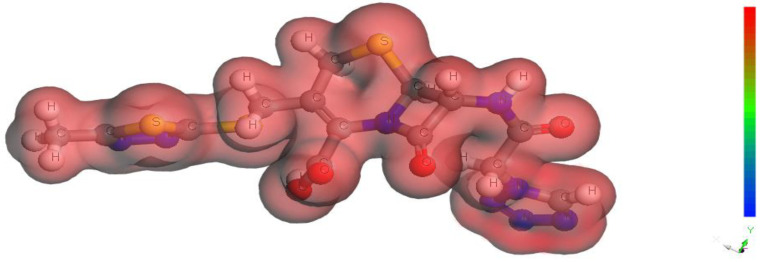
Electron density plots of the Cefazolin molecule.

Figure 11 shows the electron density plots of the Cefazolin drug. The molecular electrostatic potential (MEP) mapping of the drug Cefazolin indicates that the tetrazole ring is the nucleophilic center (the red region) for adsorption. This will improve the possibility of the adsorption process and create a protective film on the metal surface, increasing the effectiveness of inhibition. The electron density, which disperses throughout the entire molecule, strengthens the molecule’s adsorption intensity over the copper surface. The hydrophobic carbon chain supports the attachment of adsorbed molecules by repelling aggressive chloride anions farther from the surface [66].

Several descriptors can be calculated from the DFTB results, which assess the inhibition performance of the drug. They can be computed from the highest occupied molecular orbital energy (*E_HOMO_*) and the lowest unoccupied molecular orbital energy (*E_LUMO_*). Based on the literature [67], Table 6 reports the electronic parameters for the energy gap (Δ*E*), ionization potential (*I*), electron affinity (*A*), chemical potential (*µ*) electronegativity (*χ*), global hardness (*η*), softness (*σ*), electrophilicity index (*ω*), nucleophilicity (*ε*), and proportion of electron transfer (Δ*N_max_*). The parameters are calculated according to the following relations [12]:

(17) $$\Delta E=E_{\mathrm{LUMO}}-E_{\mathrm{HOMO}}$$

(18) $$I=-E_{\mathrm{HOMO}}$$

(19) $$A=-E_{\mathrm{LUMO}}$$

(20) μ=−χ 

(21) $$\mu =\frac{\left(E_{\mathrm{HOMO}}+E_{\mathrm{LUMO}}\right)}{2}$$

(22) $$\eta =\frac{\left(E_{\mathrm{LUMO}}-E_{\mathrm{HOMO}}\right)}{2}$$

(23) σ=1η

(24) $$\Delta N_{max}=\frac{-\mu }{\eta }$$

(25) $$\omega =\frac{\mu^{2}}{2\eta }$$

(26) ε=1ω

The ionization potential of molecules expresses their chemical reactivity. Smaller ionization energy values indicate greater reactivity of the atoms and molecules in contrast to large values, which indicate great stability of the molecules [68]. Table 6 indicates that Cefazolin has a strong inhibitory efficiency because of its low ionization energy value.

The χ value of the Cefazolin drug is a significant factor to be considered when evaluating its reactivity. The value indicates the degree to which it may keep electrons [69]. A small value of σ and a high value of *η* confirm a significant interaction between Cefazolin and the copper metal. [14]. The electrophilicity (*ω*) value of the Cefazolin molecule demonstrates stability. The reciprocal of electrophilicity provides nucleophilicity (*ϵ*). The estimated Δ*N_max_* value for Cefazolin is 1.92, suggesting that it can release electrons, confirming the formation of a protective film on the copper surface [70]. According to quantum simulations, the current inhibitor has effective adsorption sites that can easily adsorb on the copper surface and form a protective film.

#### 2.7.2. Monte Carlo Simulation (MC)

**Figure 12:**
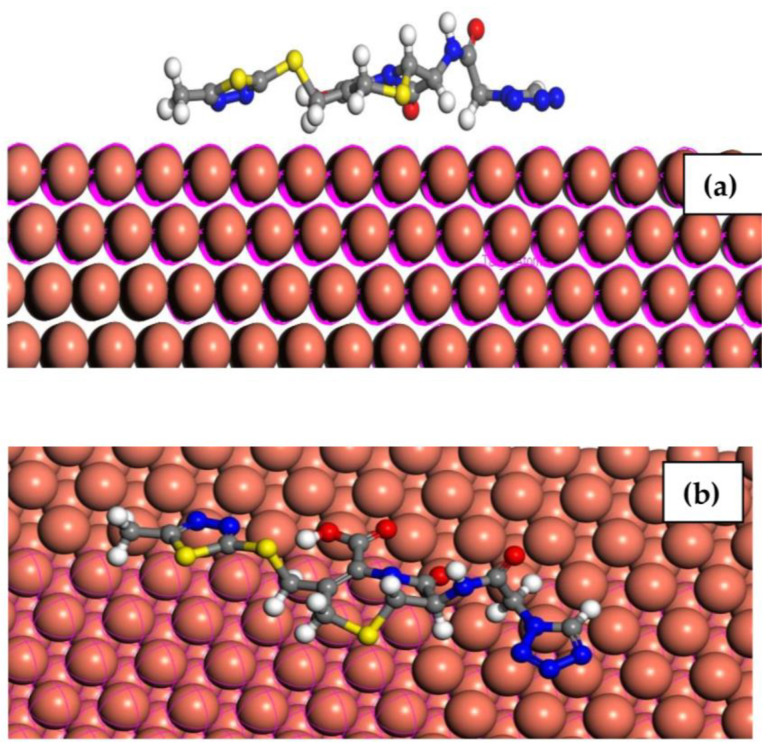
Side view (**a**) and top view (**b**) for the adsorption of the Cefazolin drug on copper surface.

Monte Carlo simulations are used to study the interaction between a single drug molecule and the copper surface. MC modeling is a widely used computational technique for simulating the adsorption behavior of molecules on solid surfaces, including corrosion inhibitors. It can provide insights into the adsorption thermodynamics and kinetics, as well as the spatial arrangement of adsorbed molecules on the surface. The MC results show that Cefazolin exhibits favorable adsorption on the metal surface with a negative adsorption energy, indicating spontaneous adsorption. Figure 12 shows the side and top views of the most stable adsorption configuration of the Cefazolin molecule on the surface of copper. It is apparent from the side view of the optimized structure (Figure 12a) that the Cefazolin drug is adsorbed parallel to the Cu (111) surface via the donation of a lone pair of heteroatoms and π–electrons of the tetrazole ring to the copper metal [71]. A top–view image of the optimized structure shows that the Cefazolin molecule highly covers the copper surface (Figure 12b).

The adsorption, deformation, and rigid adsorption energies are calculated as shown in Table 7. The adsorption energy, *E_ads_*, is calculated according to Equation (27).

*E_ads_* = *E_Cu−inh_* − *(E_inh_* − *E_Cu_)* (27)

where *E*_inh_, and *E*_Cu_ are the total energy of the inhibitor and the copper surface, respectively. The Cu(111) surface energy is assumed to be zero [72]. The adsorption energy is the energy evolved or absorbed when the relaxed drug molecule is adsorbed on the copper surface. It is the sum of the rigid adsorption energy and the deformation energy of the drug molecule. The rigid adsorption energy reports the energy released or absorbed when the unrelaxed drug molecule is adsorbed on the copper. The deformation energy is the energy released when the adsorbed adsorbate molecule is relaxed on the copper surface [72]. The adsorption energy is found to be −459.38 kJ mol^−1^. The negative *E_ads_* value indicates that the adsorption is an energetically feasible process. A high adsorption energy value suggests that the Cefazolin molecule is a possible efficient inhibitor.

### 2.8. Mechanism of Corrosion Inhibition

**Figure 13:**
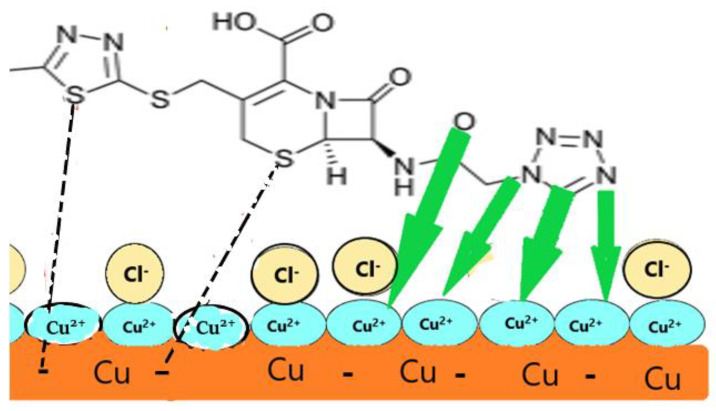
Proposed mechanism of the Cefazolin as corrosion inhibitor for copper metal.

According to Table 2, the calculated values of Δ*G^o^_ads_* are −12.81 to −16.68 kJ mol^−1^, indicating that physisorption is the process by which the examined drug binds to copper metal in a solution of 0.5 mol L^−1^ HCl. This result is supported because inhibition efficiency decreases as the temperature increases. The presence of N, O, and π–electrons in the structure of the examined drug may be responsible for its interaction with the copper surface. According to Equations (3) and (7), O_2_ and H^+^ in the solution are mixed to form water at the cathode, while Cu metal is oxidized to Cu^+^ at the anode. In addition, the Cefazolin molecule accepts electrons from the copper metal through the dithiazole ring and 5–thia–1–azabicyclo[4.2.0]oct–2–ene–2–carboxylic acid. The MC results confirm this. The proposed mechanism of inhibition is illustrated in Figure 13.

According to the values of *E_corr_*, *β_a_*, and *β_c_* in Table 3, the addition of the Cefazolin drug to the corrosive medium affects not only the reduction of oxygen gas in the cathodic area but also the oxidation of Cu metal in the anodic area. Through physisorption, the Cefazolin molecules and the chloride ions in the corrosion environment create a protective film on the Cu surface. This film reduces the metal contact area with the corrosive medium and suppresses additional Cu^+^ oxidation. According to Xu et al. [73], the rich electronic center (N and conjugate rings) of Cefazolin molecule and the Cu^+^ ions share the charge and generate the active complex to inhibit the corrosion reaction.

### 2.9. A Comparison between the Expired Cefazolin Drug and Other Drugs Previously Mentioned 

Table 8 compares the examined expired Cefazolin drug with other drugs described throughout the literature for corrosion inhibition. The comparison shows that the expired Cefazolin drug is more efficient than many expired drugs at inhibiting corrosion.

## 3. Materials and Methods

### 3.1. Cu Coupons

The composition (weight%) of the copper metal used in this study was determined by EDX analysis and was found to be 0.031 iron, 0.004 silicon, 0.022 lead, 0.011 nickel, and the rest was Cu.

### 3.2. Chemicals and Solutions

The Cefazolin structure, IUPAC nomenclature, molecular mass, and molecular formula are shown in Table 9. The employed expired Cefazolin, HCl acid solution, and ethyl alcohol (C_2_H_5_OH) were purchased from Merck Company of pharmaceuticals (Cairo, Egypt) and used directly.

An amount of 1 g of an investigated expired antibiotic was accurately dissolved in 1 L of doubly distilled water to form a stock solution with a concentration of 1000 parts per million (ppm). The stock solution was then diluted to the desired concentration (50–300 ppm) with the appropriate amount of doubly distilled water. High–grade HCl (32 wt.%) was purchased from Sigma-Aldrich (St. Louis, MO, USA). It was diluted with doubly distilled water to prepare the corrosive medium, 0.5 mol L^−1^ HCl.

### 3.3. WL Method

WL measurements were established according to ASTM–G1 [82]. For twenty–four hours at 298–318 K, copper sheets with dimensions of (2 × 2 × 0.1 cm^3^) were immersed in 0.5 mol L^−1^ HCl without and with different doses of the drug. They were then cleaned, dried, and weighed.

### 3.4. Electrochemical Measurements

The electrochemical measurements were performed according to ASTM–G102. For PP measurements, a conventional cell with a platinum electrode (counter) and saturated calomel (SCE) as a reference electrode was used [13]. The working Cu rod electrode was pressure–fitted into a polytetrafluoroethylene (PTFE) holder, exposing a surface area of 1 cm^2^ to the corrosive medium. The unmasked surface was polished with the emery cloth, cleaned with acetone and distilled water, and then left to dry. An air thermostat was used to take the readings at a constant temperature. By adjusting the copper potential from −0.7 to −0.2 V vs. SCE, the polarization graphs were plotted. A scan rate of 0.2 mV s^−1^ was utilized. Prior to each run, the copper electrode was immersed in the blank solution for 30 min at open circuit potential (OCP) to obtain a steady state.

EIS data were gathered using a sine wave of 10 mV over a wide frequency limit of 100 kHz to 5 × 10^−4^ Hz. A corrosion program was connected to Volta Lab 40 Potentiostat PGZ 301 for measuring. The copper electrode was fixed at the OCP for 30 min in the examined solution before the impedance spectra were recorded. Two frequencies, 2 and 5 Hz, were used for the EFM study. The waveform renewed at the lowest frequency of 0.1 Hz every second. Current responses associated with harmonic current peaks and inter–modification current peaks were included in the spectrum [83]. The larger peaks were used to calculate the Tafel slopes (*β_c_* and *β_a_*), corrosion current, and causality factors (CF–2 and CF–3).

### 3.5. Surface Examination

The copper coupons were immersed in a blank solution containing 300 ppm of the expired drug for 8 h to create the adsorbed film. The coupons were then withdrawn and dried. Both SEM and EDX techniques were used to analyze the surface film. Joel JSM–6510 LV SEM was used to test the metal surface after 8 h of immersion in a 0.5 mol L^−1^ HCl solution without and with the drug. The elemental analysis of the copper surface was performed using EDX technique (EDX–FEI–QUANTA FEG 250).

### 3.6. Computational Studies

A simulation explained the mechanism of adsorption using quantum chemistry. The quantum chemical features were related to the stated inhibitory effectiveness of the tested corrosion inhibitor. DFTB +1.3 was used in the quantum calculation package. The empirical parameters used for integral evaluation in the DFTB calculation were taken from Slater–Koster file libraries. Auorg parameters were used for the present system [84,85,86]. The IE of Cefazolin was evaluated by calculating several electronic and thermodynamic parameters, such as the adsorption energy, the energy of the highest occupied molecular orbital (*E_HOMO_*), the energy of the lowest unoccupied molecular orbital (*E_LUMO_*), and the energy gap (Δ*E*).

A Monte Carlo (MC) simulation investigated the preferential adsorption configurations of Cefazolin molecules on adsorption sites of the periodic structure of copper. Possible adsorption sites were identified by carrying out MC searches of the possible substrate–adsorbate configurations using a simulated annealing algorithm [84]. MC was performed in a periodic slab model box of super unit cells with a vacuum layer of 20 Å to avoid interaction between periodic unit cells. A single–molecule inhibitor covered a super cell of a copper (111) surface with 9 atomic layers. The compass force field was used to describe the behavior of the Cefazolin molecule on the copper surface during adsorption, as well as the mechanism of interactions involving the molecule and the metal surface.

## 4. Conclusions

The expired Cefazolin drug effectively inhibited the corrosion of copper metal in 0.5 mol L^−1^ HCl. Potentiodynamic polarization, electrochemical frequency modulation, weight loss, and EIS techniques were used to evaluate the performance of the Cefazolin drug as a protective film. There was good agreement between the weight loss method and the potentiodynamic polarization, electrochemical frequency modulation, and EIS techniques. In 0.5 mol L^−1^ HCl media, Cefazolin showed remarkable inhibition efficiency for copper. The anticorrosion performance enhanced with increasing the concentration of Cefazolin drug, attaining 87% efficiency at 300 ppm, although it decreased at higher temperatures. The inhibition was mainly caused by the adsorption of the expired drug on the copper surface. The adsorption matched the Temkin model. The thermodynamic parameters suggested the physisorption of the drug, forming a protective film on the copper surface. Cefazolin drug is a mixed–type inhibitor. The micrographs and spectra obtained from the SEM and EDX analyses, respectively, supported these findings. DFTB and MC simulation results indicated that the expired drug highly bound to the copper surface via the lone pair of electrons on the heteroatoms, in addition to the π–electrons of the tetrazole ring. The adsorption energy between the drug and copper surface was −459.38 kJ mol^−1^.

## Figures and Tables

**Table 1:** Data of WL measurements at 180 min for copper in 0.5 mol L^−1^ HCl, in the absence and presence of different concentrations of expired Cefazolin drug at different temperatures.

Temperature (K)	Dose of Drug (ppm)	Weight Loss (mg cm^−2^)	*k* × 10^−3^ (mg cm^−2^ min^−1^)	Surface Coverage *θ*	*IE_w_* (%)
298	0.00	8.50	11.8	–	–
	50	3.5	4.86	0.59	59 ± 0.2
	100	3	4.16	0.65	65 ± 0.1
	150	2.7	3.75	0.68	68 ± 0.3
	200	1.9	2.64	0.78	78 ± 0.2
	250	1.5	2.08	0.82	82 ± 0.3
	300	1.1	1.50	0.87	87 ± 0.2
303	0.00	9.1	12.6	–	–
	50	3.7	5.13	0.53	53 ± 0.3
	100	3.3	4.58	0.64	64 ± 0.5
	150	3.1	4.30	0.66	66 ± 0.4
	200	2.2	3.05	0.76	76 ± 0.2
	250	1.65	2.28	0.82	82 ± 0.1
	300	1.3	1.81	0.86	86 ± 0.2
308	0.00	11	15.2	–	–
	50	5.3	7.36	0.52	52 ± 0.5
	100	4.2	5.83	0.62	62 ± 0.6
	150	3.9	5.41	0.65	65 ± 0.1
	200	2.9	4.03	0.74	74 ± 0.3
	250	2.3	3.19	0.79	79 ± 0.3
	300	1.9	2.63	0.83	83 ± 0.2
318	0.00	16.5	22.9	–	–
	50	8.43	11.7	0.49	49 ± 0.3
	100	7.34	10.2	0.56	56 ± 0.1
	150	6.33	8.79	0.62	62 ± 0.2
	200	5.28	7.33	0.68	62 ± 0.5
	250	4.67	6.48	0.61	61 ± 0.2
	300	3.55	4.93	0.78	78 ± 0.4

**Table 2:** Adsorption parameters for copper at various concentrations of expired Cefazolin at different temperatures in 0.5 mol L^−1^ HCl solution.

Temperature (K)	*K_ads_* (L mol^−1^)	−Δ*G^o^_ads_* (kJ mol^−1^)
298	15.14	16.68
303	7.59	15.22
308	3.55	13.53
313	2.82	13.15
318	2.29	12.81

**Table 3:** PP data of copper in 0.5 mol L^−1^ HCl containing different concentrations of expired Cefazolin at 298 K.

Concentration (ppm)	−*E_corr_* × 10^−3^ (V/SCE)	*i_corr_* × 10^−6^ (A cm^−2^)	*β_a_* × 10^−3^ (V dec^−1^)	−*β_c_* × 10^−3^ (V dec^−1^)	*IE_p_* (%)
Blank	420	542	115	136	_
50	415	240	122	125	55.7 ± 0.1
100	403	212	116	133	60.9 ± 0.3
150	395	165	107	118	69.6 ± 0.3
200	388	134	114	127	75.2 ± 0.2
250	380	108	105	134	80.1 ± 0.2
300	372	78	98	119	85.6 ± 0.1

**Table 4:** EIS data of copper in 0.5 mol L^−1^ HCl in the absence and presence of different concentrations of expired Cefazolin at 298 K.

Concentration (ppm)	*C_dl_* × 10^−6^ (F cm^−2^)	*R_ct_* (Ω cm^2^)	*θ*	*IE_s_* (%)
Blank	343	55.4	_	–
50	163.5	130.7	0.58	58 ± 0.3
100	155.1	170.5	0.77	77 ± 0.3
150	142.4	220.3	0.75	75 ± 0.4
200	104.8	270.8	0.80	80 ± 0.1
250	88.7	355.6	0.84	84 ± 0.2
300	68.9	487	0.89	89 ± 0.2

**Table 5:** Electrochemical kinetic parameters obtained by EFM technique for copper metal in 0.5 mol L^−1^ HCl in the absence and presence of different concentrations of expired Cefazolin at 298 K.

Concentration (ppm)	*i_corr_* × 10^−6^ (A cm^−2^)	*β_a_* × 10^−3^ (V dec^−1^)	−*β_c_* × 10^−3^ (V dec^−1^)	CF–2	CF–3	*IE_EFM_* (%)
Blank	542	112	124	1.95	3.09	–
50	230	122	133	2.02	3.03	57.6 ± 0.2
100	206	113	215	1.98	2.98	61.2 ± 0.3
150	160	126	186	1.94	2.89	70.4 ± 0.1
200	128	98	154	2.08	3.06	76.4 ± 0.2
250	105	114	164	2.01	3.04	80.6 ± 0.2
300	80	99	145	1.96	2.93	85.1 ± 0.3

**Table 6:** The calculated quantum chemical parameters obtained from DFT theory for Cefazolin molecule.

HOMO (au)	LUMO (au)	∆*E* (au)	*I* (au)	*A* (au)	*η* (au)	*σ* (au)^−1^	*µ* (au)	*χ* (au)	*ω* (au)	*ε* (au)	Δ*N_max_* (au)
−6.40	−2.01	4.39	6.4	2.01	2.19	0.46	−4.21	4.21	4.04	0.25	1.92

**Table 7:** The descriptors calculated by the Monte Carlo simulation for the adsorption of the Cefazolin drug on the Cu surface.

Total Energy (kJ mol^−1^)	Adsorption Energy (kJ mol^−1^)	Rigid Adsorption Energy (kJ mol^−1^)	Deformation Energy (kJ mol^−1^)
−598.67	−459.38	−453.32	−6.06

**Table 8:** The comparison between the tested expired Cefazolin drug and other expired drugs reported in the literature.

Drug Name	Inhibition Efficiency	References
Expired Concor	75.1% at 300 ppm	[74]
Expired Moxifloxacin	69.7% at 300 ppm	[75]
Expired Acetazolamide	81.4% at 300 ppm	[76]
Expired Phenytoin	79.0% at 500 ppm	[77]
Expired Ranitidine	89.0% at 400 ppm	[78]
Expired Doxycycline	68.5% at 200 ppm	[79]
Expired Cephapirin	83.0% at 600 ppm	[80]
Expired Irbesartan	83.0% at 300 ppm	[81]
Expired Cefazolin	87.0% at 300 ppm	This work

**Table 9:** The chemical structure, IUPAC name, molecular mass, and formula of the Cefazolin drug.

Drug	Chemical Structure	Characteristics
Cefazolin	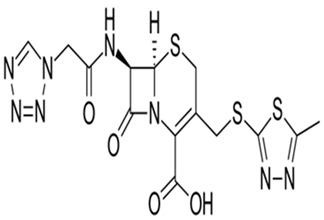	**IUPAC name:** (6*R*,7*R*)–3–{[(5–methyl–1,3,4–thiadiazol–2–yl)thio]methyl}–8–oxo–7–[(1*H*–tetrazol–1–ylacetyl)amino]–5–thia–1–azabicyclo[4.2.0]oct–2–ene–2–carboxylic acid **Molecular Formula:** C_14_H_14_N_8_O_4_S_3_ **Molecular mass:** 454.51 g/mol

## Data Availability

The data presented in this study are available on request from the corresponding author.
